# Characterization of *Amoeboaphelidium protococcarum*, an Algal Parasite New to the Cryptomycota Isolated from an Outdoor Algal Pond Used for the Production of Biofuel

**DOI:** 10.1371/journal.pone.0056232

**Published:** 2013-02-20

**Authors:** Peter M. Letcher, Salvador Lopez, Robert Schmieder, Philip A. Lee, Craig Behnke, Martha J. Powell, Robert C. McBride

**Affiliations:** 1 Department of Biological Sciences, The University of Alabama, Tuscaloosa, Alabama, United States of America; 2 Crop Protection Group, Sapphire Energy, Inc., San Diego, California, United States of America; 3 Department of Computer Science and Computational Science Research Center, San Diego State University, San Diego, California, United States of America; University of California Riverside, United States of America

## Abstract

Mass culture of algae for the production of biofuels is a developing technology designed to offset the depletion of fossil fuel reserves. However, large scale culture of algae in open ponds can be challenging because of incidences of infestation with algal parasites. Without knowledge of the identity of the specific parasite and how to control these pests, algal-based biofuel production will be limited. We have characterized a eukaryotic parasite of *Scenedesmus dimorphus* growing in outdoor ponds used for biofuel production. We demonstrated that as the genomic DNA of parasite FD01 increases, the concentration of *S. dimorphus* cells decreases; consequently, this is a highly destructive pathogen. Techniques for culture of the parasite and host were developed, and the endoparasite was identified as the Aphelidea, *Amoeboaphelidium protococcarum*. Phylogenetic analysis of ribosomal sequences revealed that parasite FD01 placed within the recently described Cryptomycota, a poorly known phylum based on two species of *Rozella* and environmental samples. Transmission electron microscopy demonstrated that aplanospores of the parasite produced filose pseudopodia, which contained fine fibers the diameter of actin microfilaments. Multiple lipid globules clustered and were associated with microbodies, mitochondria and a membrane cisternae, an arrangement characteristic of the microbody-lipid globule complex of chytrid zoospores. After encystment and attachment to the host cells, the parasite injected its protoplast into the host between the host cell wall and plasma membrane. At maturity the unwalled parasite occupied the entire host cell. After cleavage of the protoplast into aplanospores, a vacuole and lipids remained in the host cell. *Amoeboaphelidium protococcarum* isolate FD01 is characteristic of the original description of this species and is different from strain X-5 recently characterized. Our results help put a face on the Cryptomycota, revealing that the phylum is more diverse than previously understood and include some of the Aphelidea as well as *Rozella* species and potentially Microsporidia.

## Introduction

The production of biofuels using algae is an attractive technology that could mitigate the impact of climate change, the ongoing depletion of fossil reserves, and foster continued economic growth and stability [1]. There are a number of challenges to the economic production of biofuels; in particular, producing algae cost effectively at an agricultural scale, which has not yet been demonstrated [2]. Open ponds have been extensively studied and are considered to be the lowest cost and most scalable technologies for the production of algae [3], [4]. One of the hurdles impacting the implementation of cultivating algae in open pond systems is contamination by predators and fast growing heterotrophs [5]. Parasitic attacks can be devastating, destroying mass cultures in a matter of days. Unless contamination can be controlled, it is unlikely that open ponds will ever reach their potential in the production of algae for biofuel [4].

Numerous taxa in the basal fungi are primary parasites of the green algae [6] that are major players in the biofuel industry. We have been exploring eukaryotic parasites attacking open ponds of *Scenedesmus dimorphus* grown for biofuel production in New Mexico, USA. Understanding these parasites’ life histories and phylogenetic relationships will help in the development of future strategies to control attacks in outdoor algal cultivation facilities [7]. We have identified one of these parasites as *Amoeboaphelidium protococcarum* Gromov and Mamkaeva, which at the time of our identification had not been characterized phylogenetically based on gene sequence analysis. Recently, however, another organism identified as *A. protococcarum* (strain X-5) [8] from ponds at a more northern latitude (Kamchatka Peninsula, Russian Far East) [9] has been phylogenetically analyzed and placed within the Cryptomycota clade [10]. The Cryptomycota was erected based on phylogenetic analyses of gene sequences of two isolates of *Rozella* (an unwalled endoparasite of fungi and fungal-like organisms) and environmental samples [11], [12], [13]. The purposes of our study are to provide additional insights into the phylogenetic position, cultivation, and development of this plasmodial algal parasite and to compare our algal parasite isolate with that which Gromov and Mamkaeva described [14], [15] and contrast it with strain X-5 of Karpov et al. [10]. The results of our analyses demonstrate that these endoparasites of *Scenedesmus* are morphologically and molecularly more diverse than previously anticipated.

## Materials and Methods

### Outdoor Algae Growth

Outdoor algal growth of *Scenedesmus dimorphus* (UTEX 1237, University of Texas Culture Collection of Algae, http://web.biosci.utexas.edu/utex/) was assessed by tracking the ash-free dry weight of the alga over time using standard techniques [16]. The alga was grown in six 400 L outdoor ponds. Three of the ponds had actively replicating pests and three did not.

### Parasite Isolation

Samples were collected from ponds of *S. dimorphus* where microscopic evidence showed the presence of an amoeboid-like pest infecting numerous cells. Plaque plating was used to isolate the pest (our isolate FD01) by preparing ten-fold serial dilutions of the infected culture in 96-well plates. One-tenth mL of each dilution was added to 1 mL of a saturated *S. dimorphus* culture and 4 mL of 0.75% soft agar in 15 mL culture tubes. Culture tubes were mixed thoroughly and poured onto solid agar plates. Plates were placed in an acrylic box maintained at 33 C with continuous light (Utilitech Lighting 4100 K T8 light bulbs, ∼200 microEinsteins) and a CO_2_ flow rate of 0.3 L/min. Plaques were generated in approximately 5 to 7 d.

### Alga and Parasite Laboratory Culturing

After isolating a parasite it is necessary to know how to cultivate the parasite in order to understand its life history and to evaluate the impact of environmental manipulations on its life history. Here we describe basic conditions we developed that allow for the useful laboratory cultivation of this particular pest. There is no literature describing how to culture this pest in a controlled manner to allow the interrogation of the sorts of questions we have described here. Axenic cultures of *S. dimorphus* were grown to mid-log phase in modified artificial seawater media [MASM(D)]. MASM(D) was prepared by dissolving 1.0 g tris, 2.49 g magnesium sulfate heptahydrate, 1 g sodium bicarbonate, 0.6 g potassium chloride, 1.0 g sodium nitrate, 0.3 g calcium chloride dihydrate, 0.05 g potassium phosphate monobasic, and 6 mL *Closterium* Medium trace elements (1 g sodium EDTA, 0.194 g ferric chloride, 0.072 g manganese chloride, 0.021 g zinc chloride, 0.013 g sodium molybdate, and 0.004 g cobalt (II) chloride into 1 L DI H_2_O, sterilized using a Corning 0.22 µM filter system).

Optical density (OD) readings at 750 nm were taken on all cultures using 200 µl volume in a microplate reader, which were then diluted to a final OD of 0.2. Fifty mL of diluted culture was inoculated into 125 mL polycarbonate Erlenmeyer flasks with vented DuoCaps (Florida Scientific Products) and infected with individual cored plaques. Additionally, flasks of diluted culture were infected at 1% v/v with original culture samples used for the isolation process. Infected cultures and respective uninfected controls were placed on a shaker (150 rpm) in an acrylic box maintained at 33 C with continuous light (Utilitech Lighting 4100 K T8 light bulbs, ∼200 microEinsteins) and were provided 100% CO_2_ at a flow rate of 0.3 L/min. Cultures were monitored daily through cell titer and light microscopy.

### Parasite DNA Extraction, Purification, and Amplification

Fifty µL of pond sample was mixed with 50 µL of 0.25X lysis buffer in PCR tubes. The DNA lysis buffer (1X) was composed of: 50 mM Tris-HCl, pH 8.0; 200 mM NaCl; 20 mM EDTA, pH 8.0; 1.0% (v/v) SDS. The mixture was then placed in a PCR block and heated with the following steps: 95 C, 10 min; 25 C, 5 min; 95 C, 10 min; 25 C, 5 min. The primers (5′ to 3′) used were as follows: ITS1+2 forward TCCGTAGGTGAACCTGCGG [17], ITS1+2 reverse TCCTCCGCTTATTGATATGC [17], 18S forward ACCTGGTTGATCCTGCCAGT [18], 18S reverse GGGCATCACAGACCTG [18], 28S forward GTACCCGCTGAACTTAAGC [19], and 28S reverse TACTACCACCAAGATCT [17], [19]. The PCR reactions (50 µL each) contained: 10 µL 5X HF buffer (Phusion kit, New England BioLabs, Inc. [NEB]); 2 µL 10 mM dNTPs (NEB); 2 µL DMSO (Phusion kit, NEB); 5 µL 5 M Betaine; 2.5 µL 10 µM of each primer; 0.4 µL Phusion polymerase**;** 4 µL DNA template (boiled and diluted [1/20]); sterile H_2_O to 50 µL. PCR was run with the following steps: 98 C, 30 s; 40X (98 C, 10 s; 53 C, 30 s; 72 C, 30 s), 72 C, 5 min; 4 C hold. The PCR product was TOPO cloned (Life Technologies, Invitrogen) as per the manufacturer’s instructions. Colony PCR was performed on the *E. coli* colonies. A typical reaction contained: 35.8 µL sterile water; 5 µL 10× ExTaq buffer (Takara Bio, Inc.); 4 µL 2.5 mM each dNTPs; 2.5 µL 10 µM primer M13Flong; 2.5 µL 10 µM primer M13Rlong; 0.2 µL ExTaq enzyme. Fifty µL of master mix was dispensed to the appropriate number of wells of a PCR plate. Colonies were picked with a pipette tip and added to PCR mix. PCR reaction was run with the following protocol: 94 C, 2 min; 25X (94 C, 30 s; 60 C, 30 s; 72 C, 1 min), 72 C, 5 min; 4 C hold. Exonuclease I and Shrimp Alkaline Phosphatase (SAP) were used to remove excess primers and dNTPs from PCR products prior to submitting samples for sequencing. ExoSAP master mix was set up as follows: per reaction, 3.5 µL ddH_2_O; 0.625 µL 10X SAP buffer; 0.625 µL Exonuclease I; 1.25 µL SAP, 6 µL of 19 µL of corresponding PCR reaction was added, mixed by pipetting. Thermocycling conditions were (45 min total): 37 C, 30 min; 80 C, 15 min; 10 C.

### qPCR/parasite Tracking

Samples were collected for qPCR to track levels of genomic DNA of the target pest over time. Parasites were tracked using qPCR primers designed from ITS1 and ITS2 regions (FD01Forward CCACAAATCCCTGTTACAATCA, FD01Reverse TTACCTGCGTTATGCGTGTG). Pond lysate (prepared as for PCR) containing genomic DNA was diluted 1∶20 in ddH_2_O. qPCR reactions were set up for the CFX Real TIME System (Bio-Rad) in the following manner: 5 µL SsoFast EvaGreen SuperMix (Bio-Rad Cat # 172–5204), 2.6 µL diluted lysate, 2.4 µL of oligonucleotide set diluted as determined during qPCR primer optimization. The qPCR reaction was run with the following protocol: 98 C, 2 min; 40X (98 C, 1 s; 57 C, 4 s).

### Phylogenetic Sampling

To phylogenetically place the pest isolate FD01 relative to Fungi and Microsporidia, 18S/5.8S/28S rDNA sequences were downloaded from NCBI (http://www.ncbi.nlm.nih.gov/nuccore/) using the accession numbers provided in [10]. The rDNA sequence for isolate FD01 (complete 18S, ITS1-5.8S-ITS2, and partial 28S [GenBank accession number JX967274]) was added to the sampling. For placement of isolate FD01 relative to environmental samples, 18S sequences were downloaded from NCBI using accession numbers provided in [11], [12]. The 18S rDNA sequences for isolate FD01 and strain X-5 [10] were added to the sampling.

### Phylogenetic Analyses

For the primary multi-gene analysis, rDNA sequences were trimmed to remove spacer regions (ITS) and flanking genes. The 18S and 28S sequences were aligned using SINA 1.2.11 with reference databases version 111, and 5.8S sequences were aligned using Muscle 3.8.31 with default parameters [20], [21]. The alignments were manually refined using Jalview [22] and then concatenated. The resulting alignment was subjected to Bayesian phylogenetic analysis. For this, we used MrBayes [23] with four runs (nruns = 4), each with four Markov chains (nchains = 4) for 1 000 000 generations (ngen = 1000000) with a sampling frequency of 250 generations (samplefreg = 250), six substitution categories (nst = 6) and an eight-category gamma model with spatial autocorrelation between rates at adjacent sites (rates = adgamma) and a covarion-like model (covarion = yes). The Metropolis-coupled Markov chain Monte Carlo log likelihood results were compared and the first 25 000 generations were discarded as the burn in. The resulting samples of trees were then used to construct the majority-rule consensus tree. In addition, support for nodes was assessed with maximum likelihood bootstrap as implemented in RAxML 7.2.6 [24]. The phylogenetic tree was drawn using FigTree 1.3.1 (http://tree.bio.ed.ac.uk/software/figtree/). For the supplemental single gene analysis with environmental sample sequences, the same methodology was used. The Metropolis-coupled Markov chain Monte Carlo log likelihood results were compared, and the first 125 000 generations were discarded as the burn in before constructing the majority-rule consensus tree.

### Sequence Comparison

rDNA sequences of two putatively related organisms, isolate FD01 and strain X-5 [10], were compared with BioEdit [25] for sequence similarity.

### Parasite Morphology Via Light Microscopy

Microscopy was performed by loading a 10 µL sample of culture onto a microscope slide. A microscope cover glass was placed on the sample, and visualization was performed using phase contrast on an Olympus BX51 microscope equipped with an Olympus DP72 camera. Phase contrast allowed for the clearest visualization of parasite aplanospores and stages of its development. The sample was initially visualized using the 40X objective to locate cells showing symptoms of infection, including aggregation, discoloration, pigment loss, and abnormal cell morphology. The 100X objective was then used to obtain images of different life cycle stages of the pest.

### Parasite Ultrastructure Via Transmission Electron Microscopy

MASM(D) medium was inoculated with 0.5 mL of a pure culture of *S. dimorphus* and was grown for 5 d under fluorescent lighting; at 5 d the culture had an OD at 750 nm of ∼ 0.15. This prepared culture was inoculated with 0.5 ml of a moribund *S. dimorphus* culture that had been infected 7 d previously by the putative parasite FD01. The inoculated culture of *S. dimorphus* was incubated at 32°C on a platform shaker, and 1 ml aliquots containing ∼0.25 ml of algal cells were withdrawn from the culture tube at 2, 3, 4, 5, 6, 7, 8, 14, and 26 d. Each aliquot was primary fixed with 2.5% glutaraldehyde in 0.1 M sym-collidine buffer for 1 h at 21 C, washed 3 times in 0.1 M buffer, secondary fixed with 1% osmium tetroxide in 0.1 M buffer for 1 h at 21 C in the dark, and then washed one time in 0.1 M buffer and 3 times with deionized water. Following fixation the material was centrifuged at ∼3×g and then infused with molten agar. Small blocks (∼0.1–0.2 cm^3^) of agar-infused material were immersed overnight in saturated aqueous uranyl acetate at 5 C. The blocks were dehydrated in a graded acetone series (10, 30, 50, 70, 85, 95, 100, 100%) at 15 min per step, then infiltrated with EPON resin in a graded series (12%– I h, 25%– 4 h, 50%– 4 h, 75%– 8 h, 100%– 8 h, 100%– 12 h), and then polymerized for 72 h at 70 C. Embedded material was sectioned at 100 nm with a diamond knife on a Leica Ultracut microtome, and sections were collected on 300 mesh hexagonal nickel grids. Sections were oxidized in 1% periodic acid for 4 min, washed with deionized water, and post-stained with (1) saturated uranyl acetate in 70% ethanol for 10 min followed by one wash with 70% ethanol and one wash with deionized water, and (2) lead citrate in the presence of sodium hydroxide pellets for 6 min, followed by one wash with 0.1 M sodium hydroxide and one wash with deionized water. Sections were observed at 60 kV on a Hitachi 7650 transmission electron microscope (TEM).

### Parasite Identification

Parasite isolate FD01 was identified based on our evaluation of the historical record [26], [27] and comparative morphology [14], [15].

## Results

### Parasite Isolation and Culturing

**Figure 1:**
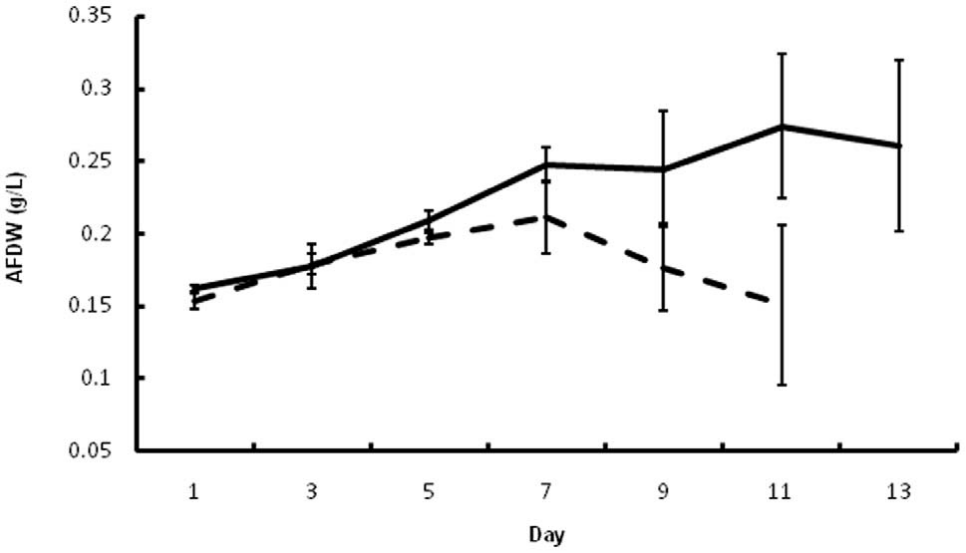
Field data on growth of *Scenedesmus dimorphus*. Ash-free dry weights (AFDW) of *S. dimorphus* ponds indicate culture productivity is reduced over time when pests are present (dashed lines **−** **−**). Ponds without pests demonstrate a continued increase in productivity (solid lines **−**). Error bars are standard deviations of three replicates.

Populations of *S. dimorphus* raised in outdoor ponds for biofuel production sometimes crash, experiencing a complete loss of productivity. Microscopic inspection of crashed cultures revealed the presence of an endoparasite, which was coded as isolate FD01. Field data indicating the devastating impact of the algal parasite FD01 on the alga *S. dimorphus* grown in outdoor ponds are shown in Figure 1. Ash-free dry weight data suggested the pest was capable of adversely affecting culture productivity over time. Ponds without actively growing pests showed no significant decrease in productivity when compared to the infected culture.

**Figure 2:**
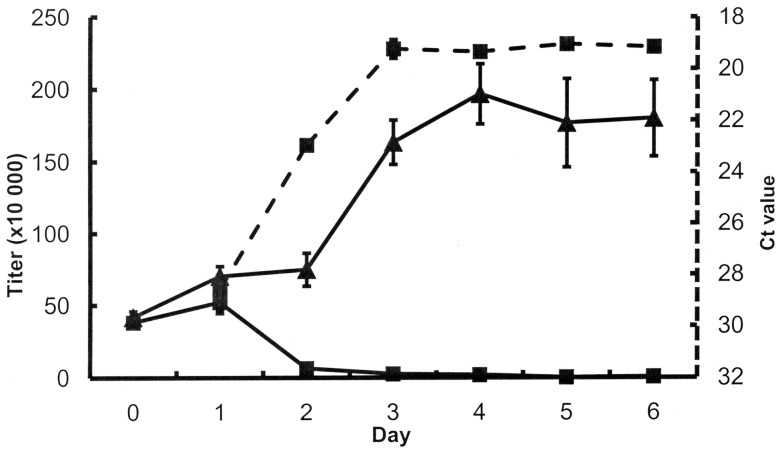
Laboratory data on growth of *Scenedesmus dimorphus*. Axenic cultures of *S. dimorphus* infected with outdoor pond culture show a decrease in cell titer over time (solid line with ▪, left Y axis [titer × 10 000]) and an increase in target pest genomic DNA (dashed line with ▪, right Y axis [Ct (cycle threshold) value]). Uninfected controls show an increase in cell titer (solid line with ▴, left Y axis [titer × 10 000]). Error bars are standard deviations of three replicates.

A key step in understanding the algal parasite was the establishment of a robust laboratory model. Figure 2 shows laboratory data of the algal pest. Samples from the diseased ponds (Figure 1) were used for pest isolation, identification, and to replicate culture infections in the laboratory. Axenic *S. dimorphus* cultures infected with pests from cultures of outdoor ponds demonstrated a decrease in cell count over time. Concurrently, qPCR confirmed that the genomic DNA of the pest increased.

### Phylogenetic Analyses

**Figure 3:**
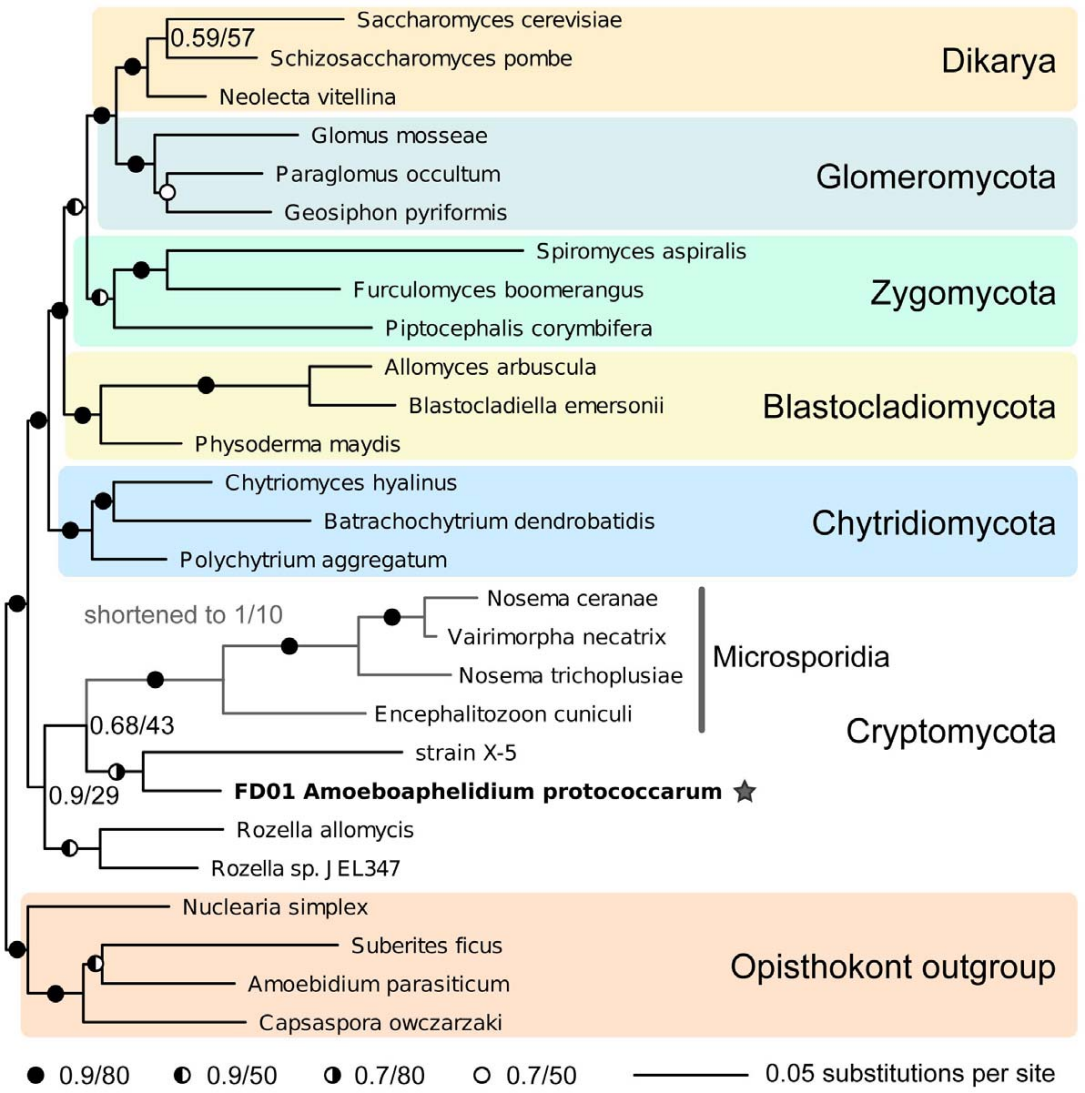
Phylogenetic placement of isolate FD01, *Amoeboaphelidium protococcarum* (star), in Cryptomycota based on multi-gene rDNA analysis. To place *A. protococcarum*, sequences from other fungal phyla and Microsporidia were included, with a Holozoa outgroup. Comparative Bayesian and ML support values are indicated. ML –lnL = 38006.62.

The phylogenetic relationship of isolate FD01 to other fungi and fungal-like organisms is illustrated in Figure 3, and sequence identifiers are included in Table S1. The relationship of isolate FD01 primarily to environmental samples is shown in Figure S1. In Figure 3, isolate FD01 was included in the monophyletic group considered as Cryptomycota [10]–[13], was sister to strain X-5, and was a relative of *Rozella* and Microsporidia. In Figure S1, isolate FD01 grouped with strain X-5 and two environmental samples; that grouping was sister to all other environmental sequences. The Microsporidia formed a monophyletic group sister to all environmental sequences.

### Sequence Comparison

rDNA sequences for isolate FD01 and strain X-5 in the Cryptomycota were compared (Table S2). The 18S of isolate FD01 had two regions (bases 1156–1522 and 1528–1651 from the 5′ end) that were absent in strain X-5. When sequences were compared without removing the regions from isolate FD01, sequence similarity was 67%; when the regions were removed, sequence similarity was 86%. The 5.8S regions had 84% sequence similarity. The 28S region of strain X-5 was complete at 3367 bases, while that of isolate FD01 was partial at 1527 bases. When the 3′ end of the strain X-5 sequence was trimmed to the 3′ end of the isolate FD01 sequence, sequence similarity was 78%.

### Parasite Morphology Via Light Microscopy

**Figure 4:**
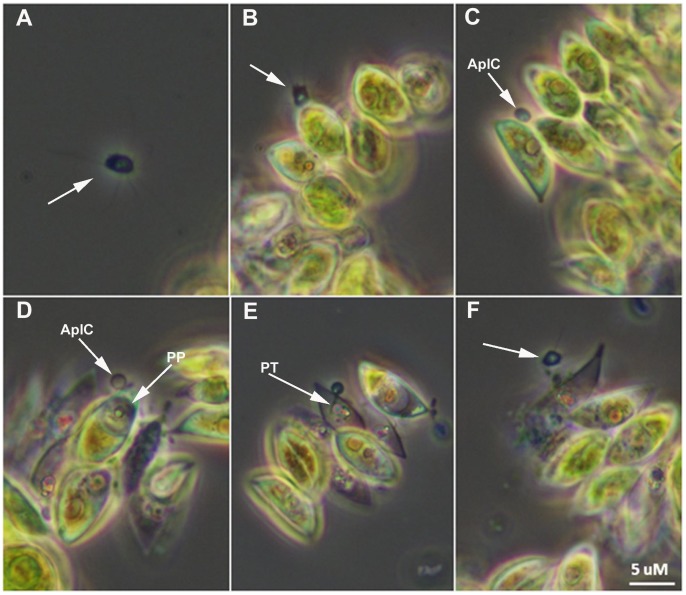
Light microscopy of life cycle of *Amoeboaphelidium protococcarum*. **A.** Motile aplanospore (arrow) proximal to host. **B.** Aplanospore (arrow) encounters *Scenedesmus dimorphus* cells and attaches. **C.** Aplanospore has completed attachment and has formed an epibiotic cyst (AplC). **D.** Parasite protoplasm (PP) has been injected into the host cell and clearly visible within the cell. **E.** Clear view of the parasite penetration tube (PT); progeny begin to mature within parasite sporangium. **F.** Dehiscence of aplanospore cyst occurs and progeny (arrow) are released from the infected cell.

Images of the pest organism FD01 across its life cycle collected using light microscopy are shown in Figure 4. Free living, motile aplanospores with several pseudopodia were evident and abundant (Fig. 4A), and were often seen proximal to host cells (HC) (Fig. 4B). As an aplanospore encountered *S. dimorphus* cells it began a phase of attachment (Fig. 4B). The attached aplanospore encysted on the host cell surface (Fig. 4C). The parasite protoplast was injected into the host cell, and the developing parasite protoplast (PP) within the cell was visible (Fig. 4D). The parasite penetration tube (PT) into the host was also visible (Fig. 4E). With the host cell wall (HCW) serving as the parasite sporangium wall, cleaved aplanospores were released (Fig. 4F) following dehiscence of a portion of the aplanospore cyst (AplC).

### Parasite Ultrastructure Via Transmission Electron Microscopy

**Figure 5:**
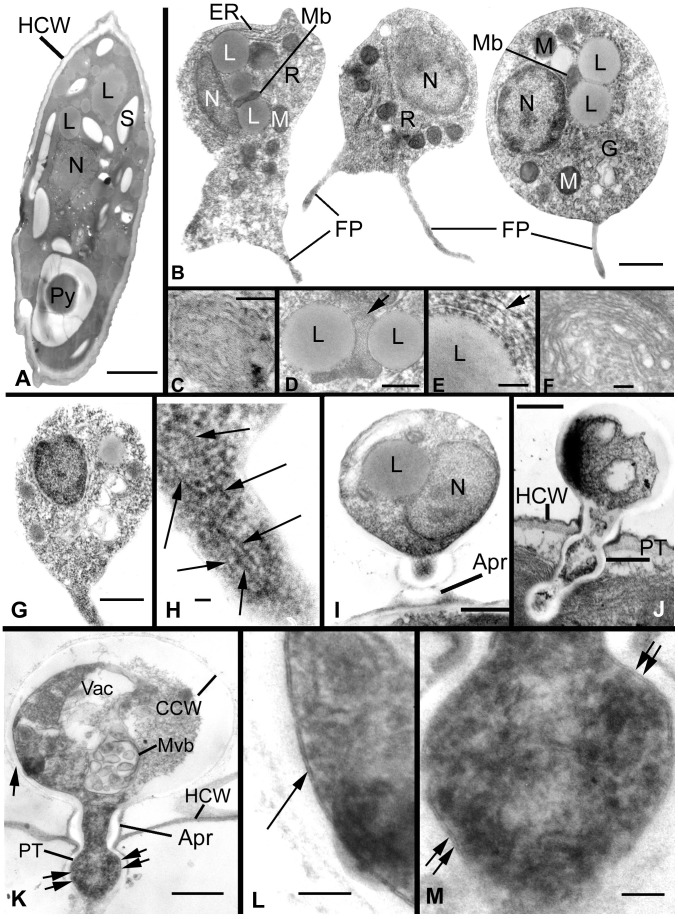
Ultrastructural features of *Amoeboaphelidium protococcarum* parasitizing *Scenedesmus dimorphus*. **A**. A healthy, uninfected *S. dimorphus* cell, surrounded by a cell wall (HCW) and containing lipid globules (L), starch granules (S), a nucleus (N), and a pyrenoid (Py). **B**. Three motile aplanospores; aplanospores have multiple filose pseudopodia (FP) and contain a single nucleus, multiple lipid globules backed by endoplasmic reticulum (ER), microbodies (Mb), multiple mitochondria (M), a Golgi apparatus (G), and dispersed ribosomes (R). **C**. Mitochondrion, illustrating lamellar cristae. **D**. Microbody (Mb) (arrow) appressed to two lipid globules. **E**. Multi-layered endoplasmic reticulum (arrow) backing microbody in the microbody-lipid globule complex. **F**. Golgi apparatus. **G**. Motile aplanospore with pseudopodium. **H**. Magnification of pseudopodium in G; putative actin microfilaments indicated by arrows. **I-K**. Aplanospore cysts. **I**. Cyst containing a nucleus and lipid globule, and subtended by an appressorium (Apr). **J**. Cyst penetrating host cell wall with a penetration tube. **K**. An aplanospore cyst surrounded by a rigid cell wall (CCW) and containing a vacuole (Vac) and a multivesicular body (Mvb); the penetration tube penetrates the host cell wall; regions indicated by single and double arrows are magnified in Figs. 3L and M. **L, M**. Single arrow (L) and double arrows (M) illustrate continuity of parasite plasma membrane around parasite protoplasm as cyst contents are injected into host (Fig. 3K). Bars: H = .025 µm; C, F, L, M = 0.1 µm; D, E = 0.25 µm; B, G, I, J = 0.5 µm; A, K = 1.0 µm.

**Figure 6:**
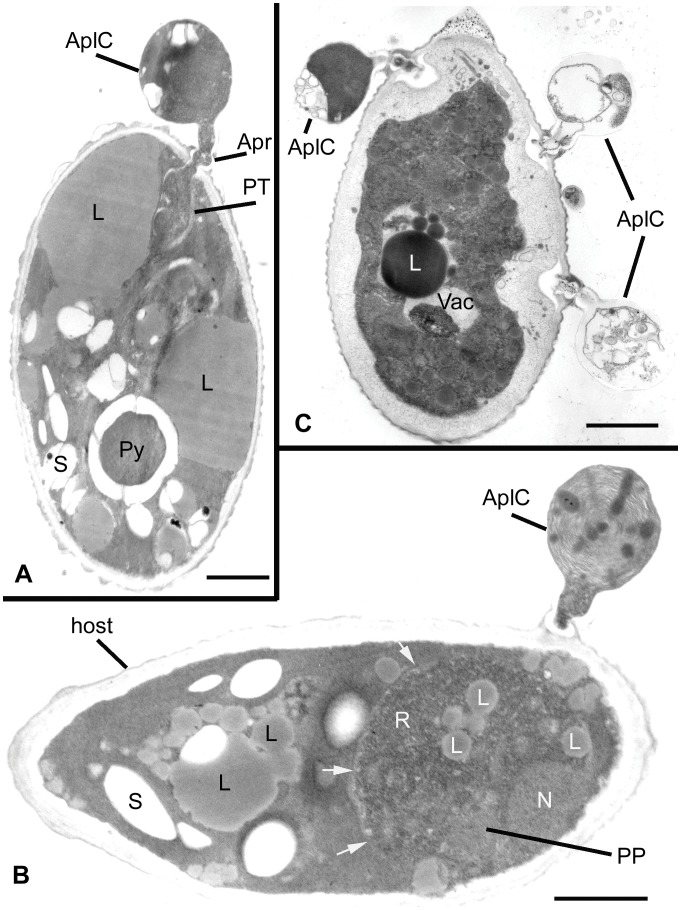
Ultrastructural features of *Amoeboaphelidium protococcarum* parasitizing *Scenedesmus dimorphus* (cont.). **A**. Early infection of host by *A. protococcarum*; aplanospore cyst subtended by an appressorium attached to host cell wall, and a penetration tube that penetrates host cell wall. **B**. Intermediate infection of host; remnant of aplanospore cyst contains myelin-like material, and developing parasite protoplasm contains ribosomes, lipid globules, and a primary nucleus; arrows indicate parasite plasma membrane. **C**. Longitudinal section of algal cell with three infections. Bars: A-C = 1.0 µm.

**Figure 7:**
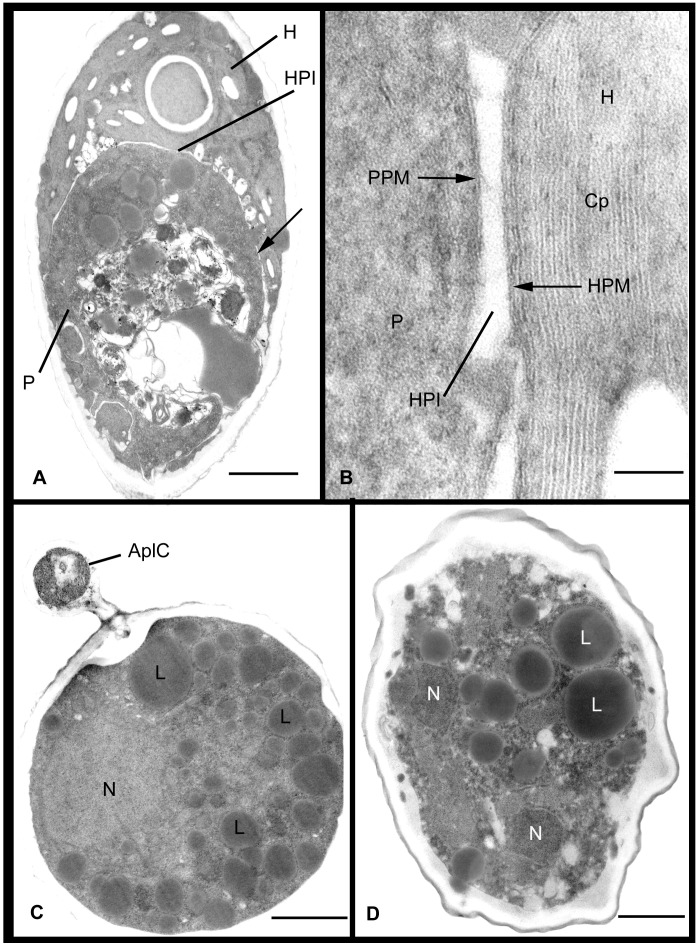
Ultrastructural features of *Amoeboaphelidium protococcarum* parasitizing *Scenedesmus dimorphus* (cont.). **A**. Advanced infection of host (H) by parasite (P), illustrating the host/parasite interface (HPI). Area indicated by arrow is enlarged in B. **B**. Host and parasite interface, illustrating opposing host plasma membrane (HPM) and parasite plasma membrane (PPM); chloroplast (Cp) indicates host location. **C**. Entire interior of infected algal cell is filled with parasite protoplast that contains a primary nucleus and multiple lipid globules. Remnant of aplanospore cyst persists on host cell wall. **D**. Multiple nuclei in mature parasite sporangium indicate the onset of mitosis. Bars: B = 0.1 µm; A, C, D = 1.0 µm.

**Figure 8:**
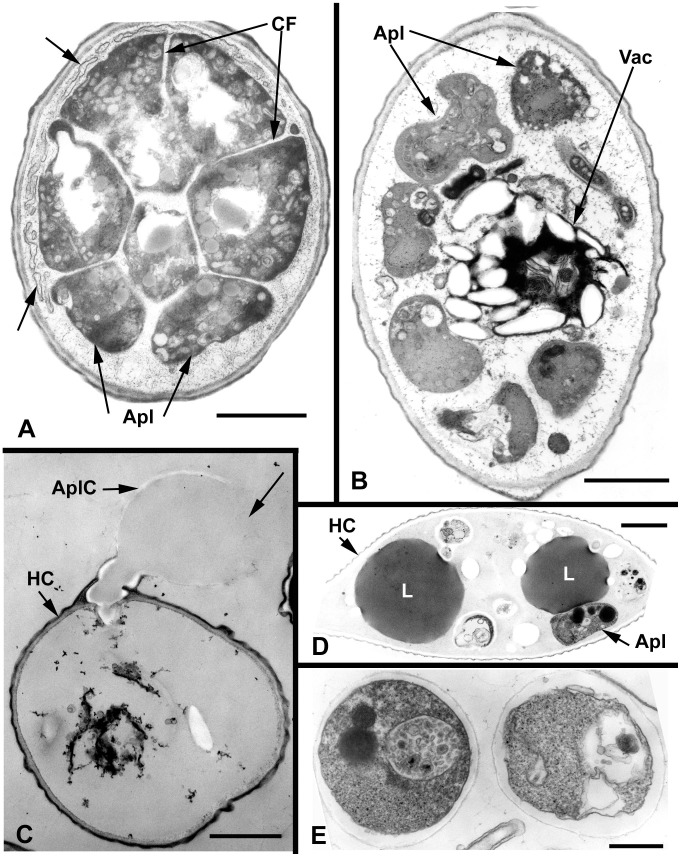
Ultrastructural features of *Amoeboaphelidium protococcarum* parasitizing *Scenedesmus dimorphus* (cont.). **A**. As indicated by cleavage furrows (CF), contents of parasite sporangium have cleaved into aplanospores (Apl); plasma membranes surrounding the sporangium have fused and fragmented (arrows). **B**. Aplanospores in host cell surround a central food vacuole. **C**. An empty host cell (HC) with an attached, empty remnant of an aplanospore cyst, of which a sub-apical portion (arrow) has dissolved. **D**. A host cell 26 d after infection, containing two large lipid globules and an unreleased aplanospore (arrow). E. Two unreleased aplanospores, each developing a thick wall. Bars: D = 0.5 µm; A, B, C = 1.0 µm; E = 0.25 µm.

**Figure 9:**
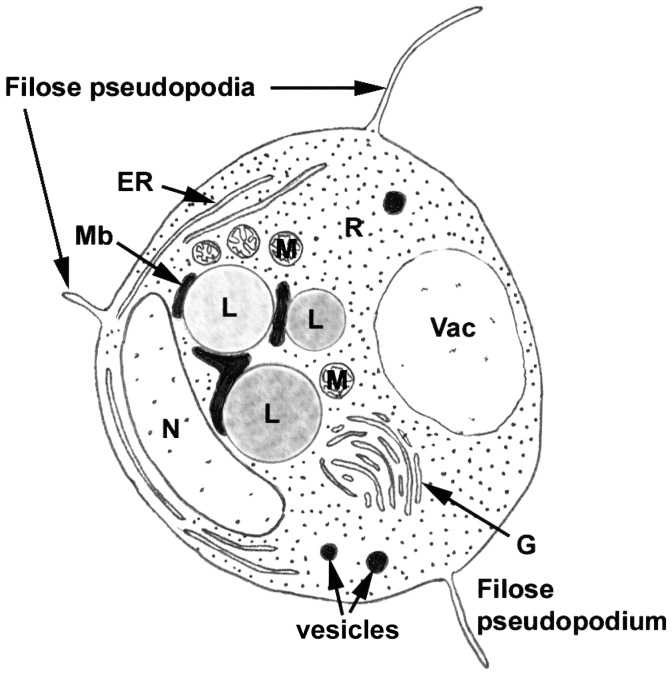
Schematic of amoeboid, filose pseudopodiate aplanospore of *Amoeboaphelidium protococcarum*, illustrating endoplasmic reticulum, Golgi apparatus, lipid globules, mitochondria, microbody, nucleus, ribosomes, vacuole, and vesicles.

**Figure 10:**
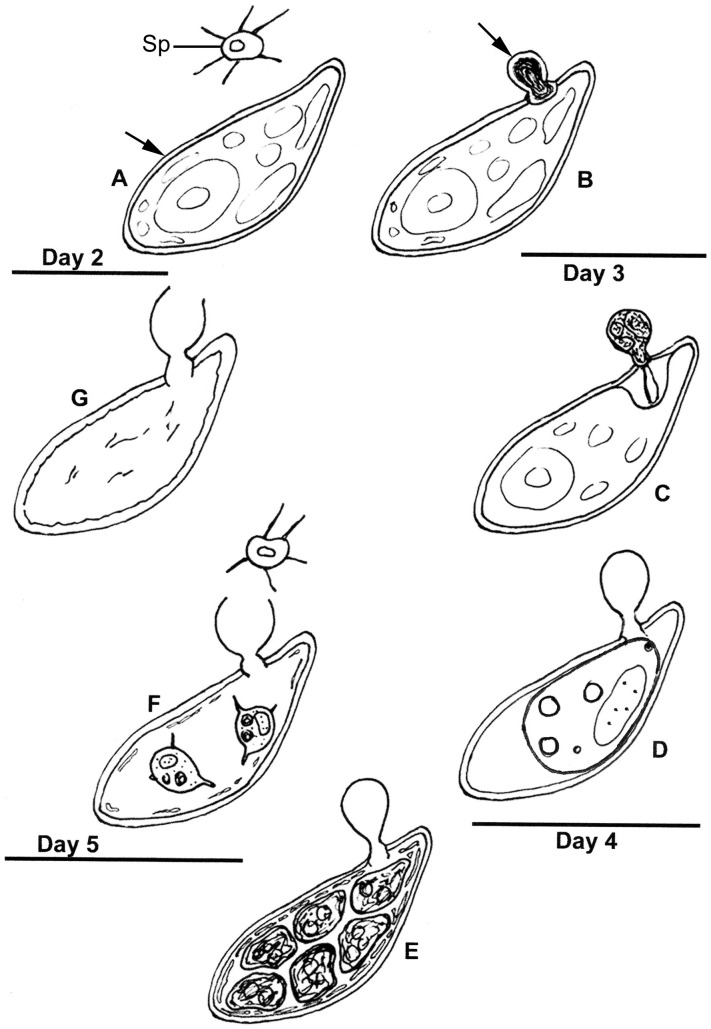
Life cycle of *Amoeboaphelidium protococcarum*. The life cycle of *A. protococcarum* is completed 3–4 days following infection. Stages of the life cycle generally correspond with Days 2–5 following infection. At **Day 2:** **A**. Abundant healthy algal cells (arrow) and abundant amoeboid, filose pseudopodiate aplanospores (Sp) are evident. **B**. A minority of algal cells indicate infection by the presence of a cyst (arrow) attached via an appressorium to the algal cell wall. **Day 3:** **C**. The walled, stalked aplanospore cyst has enlarged, a penetration tube penetrates the host cell wall, and a host reaction at the site of infection is evident. **D**. In some algal cells a membrane-bound, developing parasite protoplasm occupies a portion of the interior of the host cell, and a host/parasite interface is evident; the empty remnant of the aplanospore cyst persists. **Day 4: E**. Cleaved aplanospores within the host cell are surrounded by fused and fragmented plasma membrane; the empty remnant of the aplanospore cyst persists. **Day 5: F**. A minority of host cells retain one or a few unreleased aplanospores, a sub-apical portion of the empty remnant of the aplanospore cyst has dissolved, and abundant aplanospores occur outside host cells. **G**. Most infected host cells are empty, but retain the remnant of the aplanospore cyst.

Results of ultrastructure analysis of the pest organism FD01 are presented in Figures 5–8. In the following chronological description of the infection process, events occur generally, and may overlap, on sequential days. At Day 2, an abundance of healthy algal cells was observed (Fig. 5A), and numerous filose, pseudopodiate aplanospores were present (Fig. 5B). Aplanospores were 1.7–2.5 mµ diam, spherical, sub-spherical or elongate (Figs. 5B, 9) and contained a single nucleus (N), a microbody-lipid globule complex (MLC) consisting of multiple spherical to sub-spherical mitochondria (Figs. 5B, C) 0.25–0.5 mµ diam, with lamellar cristae, and microbodies appressed to multiple spherical lipid globules (L) (Figs. 5B, D). Multiple layers of endoplasmic reticulum (ER) backed the lipids in the MLC (Figs. 5B, E). Ribosomes (R) were dispersed in the cytoplasm (Fig. 5B), and a Golgi apparatus (Figs. 5B, F) was evident and associated with contractile vacuoles. Filose pseudopodia (FP) contained filaments of the diameter (7–8 nm) of actin microfilaments (Figs. 5G, H). At Day 3, encysted aplanospores (1.3–2 mµ diam) were found at the algal surface (Figs. 5I–K, 6A–C). A germ tube extended from the rounded infection cell, and attached to the algal surface with a globose appressorium (Apr) (Figs. 5I, K, 6A). From the appressorium, a penetration tube pierced the host cell wall and extended into the host cytoplasm (Figs. 5J, K, 6A). In the early onset of infection, the contents of the encysted aplanospore included a nucleus, one or more lipid globules, mitochondria, ribosomes, a vacuole, and a multi-vesicular body (Mvb) (Figs. 5I–K). The protoplast of the aplanospore cyst and the developing penetration tube were surrounded by a plasma membrane (Figs. 5K–M). As a host response, a plug of finely fibrillar material occasionally formed around the parasite penetration tube and between the host cell wall and plasma membrane (Figs. 5J, 4C). Some algal cells exhibited more advanced infection, in which the aplanospore cyst was vacuolated (Figs. 5H–K, 6C) or contained an abundance of myelin-like material (Fig. 6B). Following the initial stage of host infection, the parasite protoplast was injected into the host cell through the penetration tube (Figs. 5I–K, 6A). A distinct host-parasite interface was evident (Figs. 6B, 7A, B), in which the parasite plasma membrane (PPM) and investing host plasma membrane (HPM) were separated by a zone filled with finely fibrillar material (Figs. 7A, B). The parasite, thus, lay between the host cell wall and host plasma membrane. In the developing parasite protoplast, there was a large vacuole that contained oil globules, inclusions, and membranes (Figs. 8B). At Day 4, many of the infected host cells were filled entirely with the parasite, with only fragments of the host plasma membrane remaining (Fig. 8A). The host cell wall functioned as the parasite sporangium wall (Figs. 8A, B). In the sporangium, one primary nucleus (Fig. 7C) or multiple nuclei (Fig. 7D) were present. In some host cells, organized cleavage products were evident (Figs. 8A, B). After aplanospore cleavage (Fig. 8B) a large mass of host material or remnants of the parasite phagocytosis vacuole remained. At Day 5, in a minority of host cells, a few cleaved aplanospores remained (Fig. 8D). The distal, apical or sub-apical portion of the wall of the remnant of the encysted aplanospore was dissolved or dehisced (Figs. 8C). A majority of host cells were empty or contained one or several large remnant host lipid globules (Fig. 6E), occasionally an unreleased, unwalled aplanospore (Fig. 8D), and occasionally walled aplanospores (Fig. 8E). Figure 9 is a schematic of a free living, motile aplanospore. Figure 10 illustrates the life cycle, which was completed in 3–4 d and was repeated in days 5–8. At days 14 and 26, few viable algal cells remained.

### Parasite Identification

Parasite isolate FD01 was identified as *Amoeboaphelidium protococcarum* Gromov and Mamkaeva [14], [15].

## Discussion

The infection of *S. dimorphus* by *A. protococcarum* is a rapid event with devastating consequences for the algal population. Evaluation of the phylogenetic position of *A. protococcarum* and study of its life cycle via transmission electron microscopy may provide clues to mitigation or prevention of the host population crash that occurs with infection.

### Historical Record

Our evaluation of the historical record and consideration of the morphology of the organisms studied suggest that our isolate FD01 is truly *Amoeboaphelidium protococcarum*, considered as the most common parasite of protococcous algae [15], and that the flagellated strain X-5 used by Karpov et al. [10] is not *A. protococcarum*.

As a brief historical synopsis, Zopf [28] described the genus *Aphelidium*, an endoparasite of the green alga *Coleochaete*. *Aphelidium* disperses by means of posteriorly uniflagellate zoospores. Zoospores attach to a host cell, encyst, and penetrate the host via a penetration tube. The contents of the zoospore cyst flow into the host through the penetration tube, and the empty cyst wall usually remains on the host cell surface. The parasite develops as an amoeboid plasmodium that engulfs the contents of the host, growing and gradually filling the entire cell. At maturity, the plasmodium, using the wall of the host as a sporangial wall, cleaves into zoospores that subsequently leave the destroyed cell. Scherffel [26] erected the genus *Amoeboaphelidium*, which is presumably closely related to *Aphelidium*, as the two genera have similar life cycles that do not differ in the stages of vegetative growth [27]. However, *Amoeboaphelidium* forms small, motile, non-flagellated, amoeboid cells (“aplanospores”) rather than flagellated zoospores characteristic of *Aphelidium* [26], [28]–[30] and the more recently described *Pseudaphelidium* [31], [32]. For these two types of cells we use the terms “aplanospore” (a naked, amoeboid or non-amoeboid motile cell), and “zoospore” ( = “planospore”; a motile sporangiospore, i.e. one having flagella) [33].

Gromov and Mamkaeva [34] revealed an algal endoparasite described as “strains X-1 and X-2″, and later [14] described and illustrated with light micrographs *Amoeboaphelidium protococcarum* as a new species, an endoparasite of the green alga *Scenedesmus*, referring in their Material and Methods to “…Two strains of the parasites described before as X-1 and X-2 [34] have been used, new strains have been obtained from the soil surface, X-3 from Putiatin Island (Japanese Sea) and X-4 from Kamchatka” [14]. They characterized their four strains as *“…a free living parasite is a motile amoeba”…”Amoeba forms numerous pseudopodia, thin trichopodia and thick lobopodia”… ”Amoeboaphelidium is much alike Aphelidium [Fott 1957] when growing inside the host cell, but it differs from the latter in amoeba like free living stage. We have not observed flagellated cells in our strains although we examined them under different conditions during a long period of time. We can consider them only as members of the genus Amoeboaphelidium.*” [14] (our emphasis). They did not designate a particular strain as the type with the species description, and only used illustrations of strains X-1 and X-4 in the text. Thus, extensive light microscopic (LM) examinations indicate that *A. protococcarum* has a dispersal stage that is a motile amoeba with numerous pseudopodia, and is not flagellated.

Gromov and Mamkaeva [35] examined the sensitivity of different *Scenedesmus* strains to *Amoeboaphelidium*, using four strains of *A. protococcarum* (X-1, X-3, X-4, X-5). They reported that different strains of *Scenedesmus* exhibited different sensitivities to different strains of *A. protococcarum*. A reasonable conclusion from these data is that all “strains” of *A. protococcarum* may not be the same organism.

Gromov and Mamkaeva [15] described the fine structure of *Amoeboaphelidium protococcarum*, using strain X-1. In their description and illustrations there is no mention of a flagellum or flagellar apparatus. Thus, transmission electron microscopic (TEM) examinations confirm light microscopic examinations [14] and indicate *A. protococcarum* to be a non-flagellated organism.

We can only conclude from these studies that Gromov and Mamkaeva thoroughly knew the details of the morphology of *A. protococcarum* from LM examinations of strains X-1 and X-4 (and perhaps X-2 and X-3) [34] and the ultrastructure of *A. protococcarum* from TEM examination of strain X-1 [15]. We do not find in the literature any reference to morphology and/or ultrastructure of strain X-5.

Pinevich et al. [9] revealed that molecular karyotype patterns in *A. protococcarum* strains X-1 and X-5 differed. This molecular evidence indicates that strains of *Amoeboaphelidium* putatively identified as *A. protococcarum* differ genetically.

Karpov et al. [10] used strain X-5 as their representative of *Amoeboaphelidium protococcarum*, and reinvestigating the ultrastructure of the amoeboid spore found “…a pseudocilium… the permanent immotile posterior projection contains microtubules, so it may be considered as a reduced posterior flagellum, which was not described earlier” [10]. However, their examination was not of strain X-1 upon which much of the morphological configuration and the totality of ultrastructural configuration of *A. protococcarum* rests.

Our isolate FD01 fits the morphological [14] and ultrastructural [15] concepts of *Amoeboaphelidium protococcarum*: it has an amoeboid, multiple-pseudopodiate aplanospore with no evidence of flagellation, either in motile aplanospores exterior to algal cells, or in cleaved aplanospores within algal cells. Aplanospores of our isolate FD01 have filose pseudopodia that contain structural components the size of actin microfilaments (7–8 nm diameter), not microtubules (∼25 nm diameter). Conversely, Karpov et al.’s strain X-5 [10], being shown with LM (their Fig. 1e) and TEM (their Figs. 1f, g, Suppl. Fig. 1c) as posteriorly uniflagellate, does not fit the morphological concept of *A. protococcarum*. As the flagellum of strain X-5 is readily observable by both light microscopy and transmission electron microscopy, it is highly unlikely that we have overlooked this structure in our isolate FD01. Thus, on the basis of morphology and ultrastructure, we consider that our isolate FD01 and Karpov et al.’s strain X-5 [10] are not the same organism, and that our isolate FD01 is *A. protococcarum*. Because of it’s posteriorly uniflagellate condition as revealed and illustrated [10], strain X-5 may be *Aphelidium* or *Pseudaphelidium* (both having posteriorly uniflagellate zoospores), but is not *Amoeboaphelidium*.

### Morphology and Ultrastructure of *A*. *protococcarum*

Although thallus morphology and ultrastructure of *A. protococcarum* have been previously studied [14], [15], our research confirms many aspects of the life cycle and fine structure, and we also add to these observations. Here we have confirmed and illustrated via TEM observations the filose pseudopodial nature of the motile aplanospore, and revealed the presence of putative actin microfilaments in pseudopodia. We observed no flagella on free, motile aplanospores, and no flagellar sections among cleaved aplanospores, and thus do not expect this organism to have a flagellated stage.

Gromov and Mamkaeva [35]–[37] recognized various strains of *Amoeboaphelidium* that differed in their free motile stages, host specificity, and their ability to produce dormant spores. Although there was no direct evidence of resting spores with our isolate FD01, some older, senesced algal cells contained unreleased aplanospores that had developed a thick, smooth wall. Whether these cells are precursors of resting spores is speculative.

In Gromov and Mamkaeva’s [15] strain X-1 of *A. protococcarum*, at the end of sporangial development the aplanospore cyst often broke off the host cell, and aplanospore release was through the remnant of the cyst stalk. In our isolate FD01, the aplanospore cyst persisted, with a sub-apical portion dissolving, indicative of the path of spore release in isolate FD01. It is interesting that the exit orifice for the aplanospores is the same as the entrance orifice for host inoculation and infection, and that the host cell wall does not rupture or dehisce to facilitate spore release. The production of a plug around the penetration tube of the parasite was associated with the parasite attachment site. At those sites there was no evidence of successful release and injection of parasite protoplast, whereas absence of the plug was associated with successful discharge of parasite protoplast. Thus we conclude that the plug is a defense response of the host to the parasite. However, the parasite typically was able to penetrate the host prior to formation of the plug. Moreover, multiple parasite infections of individual algal cells were common, which perhaps represents a parasite strategy to overwhelm the host defense response.

### Phylogenetic Hypothesis

Our multi-gene rDNA phylogenetic hypothesis places *A. protococcarum* in Cryptomycota, a recently described phylum that branches with the Fungi [13], [14], [15]. *Amoeboaphelidium protococcarum* is also a relative of *Rozella allomycis*, *Rozella* sp., strain X-5, and Microsporidia. Our molecular analyses reinforce our contention that our isolate FD01 is not the same as strain X-5, but at the same time indicate that isolate FD01 and strain X-5 are close relatives. Our phylogenetic hypothesis is a reflection of our sequence similarity analysis of isolate FD01 and strain X-5, in which 18S, 5.8S, and 28S sequences were divergent.

Our phylogenetic hypotheses are in congruence with those of Karpov et al. [10], although our analyses used different gene sets than were used in Karpov et al. For their primary analysis of Cryptomycota and other fungal clades, Karpov et al. used rDNA (18S, 5.8S, 28S)+RPB1+RPB2 sequences for all isolates except the Microsporidia, for which rDNA sequences were excluded from analysis “…for their extremely accelerated rate of evolution” [10]. Alternatively, our primary analysis used rDNA sequences for all isolates, including the Microsporidia. Bayesian support values for the sub-tree *A. protococcarum*+*Rozella*+Microsporidia (ARM) [10] in our rDNA analysis were quite sufficient, and the groupings were the same as in Karpov et al. [10]. Although our support values are lower than those in Karpov et al., that is likely due to the additional amino acid sequences in their tree. In their supplemental (18S) analysis of the relationship of strain X-5 to environmental sequences, Karpov et al. did not include the Microsporidia, presumably for the same stated reason: the accelerated rate of evolution of Microsporidia rDNA sequences. Our supplemental analysis, examining the relationship of our isolate FD01 to primarily environmental sequences, included the Microsporidia. Again, that phylogenetic hypothesis was in congruence with that of Karpov et al. [10], and although our support values were lower than those of Karpov et al., groupings were similar. Our analyses indicate that the some Aphelidea are members of Cryptomycota, yet because of sequence divergence between *A. protococcarum* and strain X-5, much diversity has yet to be revealed.

### Environmental Considerations

*Rozella allomycis*, *A. protococcarum*, and strain X-5 are the only organisms in Cryptomycota that have been identified and described, the rest of the clade being forms of life known only by phylotypes and nucleic acid probing techniques of environmental samples [13], [14]. The 18S sequence that paired with FD01 in Figure S1 was kor_110904_24 (GenBank Accession #: FJ157332). This phylotype was recovered from environmental samples of Lake Koronia, Greece, and identified as fungal and sister of chytrids but not affiliated with any known species. Rather related sequences were derived from soils, many from extreme environments. Thus, it is possible that soils harbor the parasite *A. protococcarum* and serve as a reservoir for infection. Mitigation might include screening algal pools from airborne particulates.

### Nutritional Considerations

Our observations demonstrate that *A. protococcarum* has life cycle similarities with *Rozella allomycis* [38]–[40], which is a relative of *A. protococcarum* in our phylogenies. Gromov and Mamkaeva [15] proposed phagocytosis as the mode of nutrition for *A. protococcarum*, in which large food vacuoles captured chromatophores and other host organelles. Digestive processes occurred in a central vacuole, where the remains of digestion formed a solid excretory globule. In a TEM study of *Rozella polyphagi*, Powell [41] illustrated host mitochondria inside a vesicle in the *Rozella* thallus, and proposed phagocytosis as a means of nutrient uptake. Evidence of phagotrophic nutrition in *A. protococcarum* and the related genus *Rozella*, suggests control strategies that interfere with phagocytosis.

### Broad Phylogenetic Considerations

As in the mitochondria of *R*. *allomycis* [40] and *R. polyphagi* [41], our examination indicates that the mitochondria of *A. protococcarum* have lamellar (flat) cristae. Our evaluation of *A. protococcarum* mitochondrial morphology is in opposition to that of Pinevich et al. [9], who concluded that *A. protococcarum* mitochondria possessed tubular cristae. They hypothesized that the presence of tubular cristae, in conjunction with *A*. *protococcarum’s* nutritional mechanism of phagotrophy and its molecular relatedness with choanoflagellates (their unpublished data), were evidence of “…a specific bearing of *A. protococcarum* to primordial eukaryotic evolution”. We have demonstrated that *A. protococcarum* has lamellar mitochondrial cristae, a typical feature consistent with the Adl et al. [42] classification of the Aphelidea in the Opisthokonta.

Knowledge of this parasite’s life history, along with the knowledge that it is aligned with fungi is critical to developing an effective strategy to manage this pest in open pond cultures. Possible strategies can be evaluated to target specific stages of the life history; these strategies can be examined in lab models of this organism and then transferred to field conditions once efficacy has been determined. This understanding also allows effective treatments to be mechanistically dissected, which is useful in understanding the long term efficacy of potential strategies. Without effective pest management strategies that work in both the short and long term, the promise of biofuels at scale from algae in open pond systems becomes less tangible.

## Supporting Information

Figure S1 **Phylogenetic placement of isolate FD01,** ***Amoeboaphelidium protococcarum*** **(star), in presumptive Cryptomycota (arrow), which includes environmental sequences,** ***Rozella*** **spp., Aphelids, and Microsporidia.** To place *A. protococcarum*, sequences from other fungal phyla were included, with an Opisthokont outgroup. Comparative Bayesian and ML support values are indicated. ML –lnL = 35395.70.
(TIF)

Table S1 Sequence identifiers for isolates used in Figure 3.
(PDF)

Table S2 Comparison of gene regions in rDNA sequences of isolate FD01 and strain X-5 in Cryptomycota.
(PDF)
