# Supplementary figures and images for: Characterization of Amoeboaphelidium protococcarum, an Algal Parasite New to the Cryptomycota Isolated from an Outdoor Algal Pond Used for the Production of Biofuel
Source: PLoS One. 2013 Feb 20;8(2):e56232. doi: 10.1371/journal.pone.0056232 (PMC3577820; doi:10.1371/journal.pone.0056232)

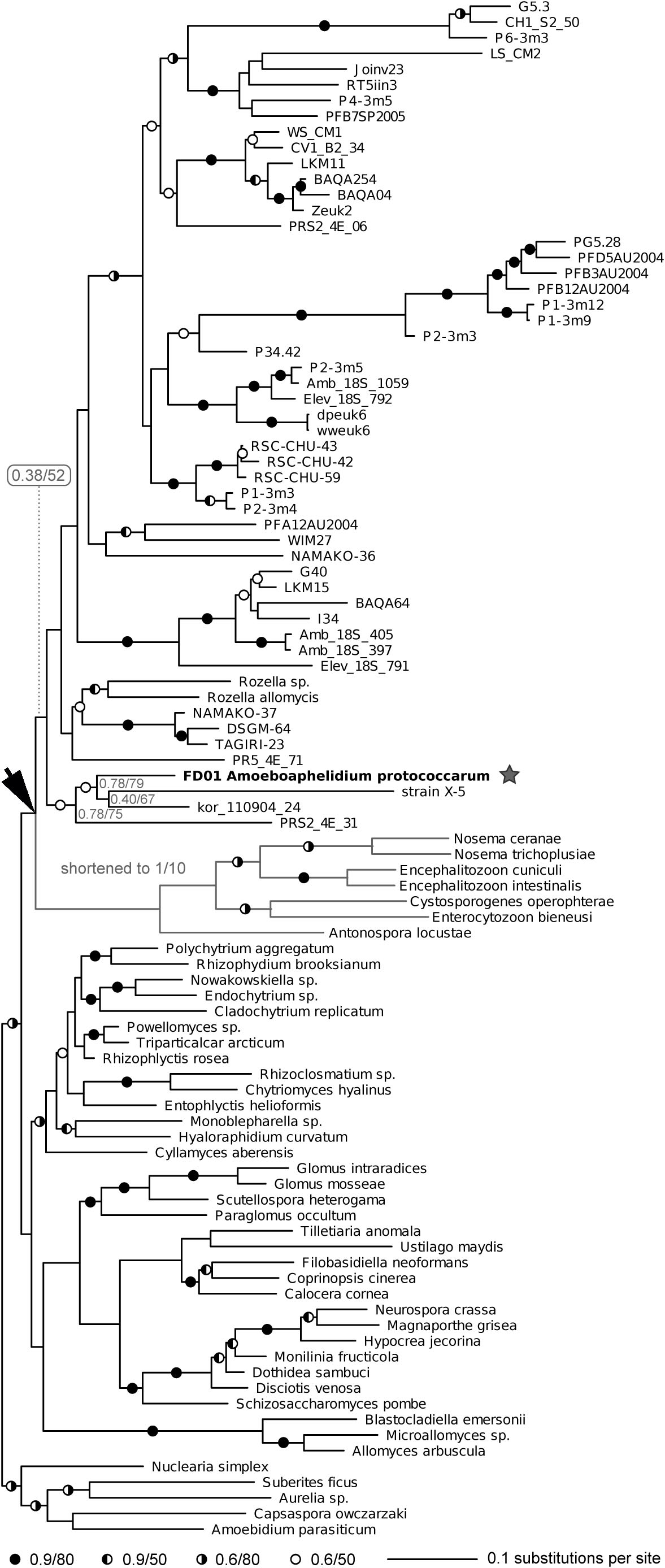

Supplement: Figure S1 — Phylogenetic placement of isolate FD01, Amoeboaphelidium protococcarum (star), in presumptive Cryptomycota (arrow), which includes environmental sequences, Rozella spp., Aphelids, and Microsporidia. To place A. protococcarum, sequences from other fungal phyla were included, with an Opisthokont outgroup. Comparative Bayesian and ML support values are indicated. ML –lnL = 35395.70. (TIF) [file pone.0056232.s001.tif]
