# Supplementary material for: Characterization of Amoeboaphelidium protococcarum, an Algal Parasite New to the Cryptomycota Isolated from an Outdoor Algal Pond Used for the Production of Biofuel
Source: PLoS One. 2013 Feb 20;8(2):e56232. doi: 10.1371/journal.pone.0056232 (PMC3577820; doi:10.1371/journal.pone.0056232)
Supplement: Table S1 — Sequence identifiers for isolates used in Figure 3. (PDF) [file pone.0056232.s002.pdf]

Supporting Information Table S1. Sequence identifiers for Figure 3.

Species	SSU	5.8S	LSU
Rozella allomycis	AY635838.1	AY997087.1	DQ273803.1
Rozella sp. JEL347	AY601707.1	AY997086.1	DQ273766.1
strain X-5	JX507298.1	JX507298.1	JX507298.1
Saccharomyces cerevisiae	Z75578.1	1360587	AY048154.1
Schizosaccharomyces pombe	288694	288694	288694
Neolecta vitellina	Z27393.1	FJ171855.1	NG_027573.1
Glomus mosseae	AY635833.1	AY997053.1	NG_027652.1
Paraglomus occultum	DQ322629.1	AY997069.1	DQ273827.1
Geosiphon pyriformis	AM183923.1	AM268204.3	AM183920.2
Spiromyces aspiralis	AF007543.1	AY997090.1	DQ273801.1
Furculomyces boomerangus	AF277013.1	AY997050.1	DQ273809.1
Piptocephalis corymbifera	AB016023.1	AY997073.1	AY546690.1
Allomyces arbuscula	AY552524.1	AY997028.1	AY552525.1
Blastocladiella emersonii	EF014366.1	AY997032.1	2440036
Physoderma maydis	AY601708.1	AY997072.1	DQ273768.1
Chytriomyces hyalinus	M59758.1	DQ536499.1	DQ273836.2
Polychytrium aggregatum	AY601711.1	AY997074.1	AY546686.1
Batrachochytrium sp.	AF051932.1	AY997031.1	AY546693.1
Encephalitozoon cuniculi	3426080	3426080	3426080
Nosema trichoplusiae	U09282.1		DQ996243.1
Vairimorpha necatrix	DQ996241.1	EU544673.1	DQ996242.1
Nosema ceranae	DQ078785.1	EF091879.1	DQ078785.1
