# Supplementary material for: Characterization of Amoeboaphelidium protococcarum, an Algal Parasite New to the Cryptomycota Isolated from an Outdoor Algal Pond Used for the Production of Biofuel
Source: PLoS One. 2013 Feb 20;8(2):e56232. doi: 10.1371/journal.pone.0056232 (PMC3577820; doi:10.1371/journal.pone.0056232)
Supplement: Table S2 — Comparison of gene regions in rDNA sequences of isolate FD01 and strain X-5 in Cryptomycota. (PDF) [file pone.0056232.s003.pdf]

Supporting Information Table S2. Comparison of gene regions in rDNA sequences of isolate FD01 *Amoeboaphelidium protococcarum* and strain X-5 (Karpov et al. 2012).

	strain X-5	isolate FD01	Sequence similarity
rDNA	1-5774 bases	1-4667 bases	
Ø region	1-98		
18S	99-1890	1-2271	67%/86%[1]
ITS1	1891-2086	2272-2630	35%
5.8S	2087-2241	2631-2798	84%
ITS2	2242-2407	2799-3140	30%
28S	2408-5774	3142-4667	78%[2]

[1] The 18S region of isolate FD01 has regions (bases 1156-1522 and 1528-1651 from the 5′ end)) that are absent in strain X-5. When sequences are compared without removing the regions, sequence similarity is 67%; when the regions are removed, sequence similarity is 86%.

[2] The 28S region of strain X-5 is complete, at 3367 bases; the 28S region of isolate FD01 is partial, at 1527 bases. When the 3’ end of the strain X-5 sequence is shortened to the length of the isolate FD01 sequence, similarity is 78%.
